# Biliary Cystadenoma: An Unusual Cause of Acute Pancreatitis and Indication for Mesohepatectomy

**DOI:** 10.1155/2014/643032

**Published:** 2014-11-18

**Authors:** Bilal Munir, Michael Meschino, Ashley Mercado, Roberto Hernandez-Alejandro

**Affiliations:** London Health Sciences Centre, 339 Windermere Road, London, ON, Canada N6G 2V4

## Abstract

The classic presentation of cystic hepatobiliary lesions is usually nonspecific and often identified incidentally. Here we describe the case of a patient presenting with acute pancreatitis resulting from a large centrally located biliary cystadenoma compressing the pancreas. Determination of the origin of the cystic lesion was difficult on imaging studies. Due to the difficult location of the lesion, a complete surgical resection was achieved with mesohepatectomy and the suspected diagnosis confirmed by pathology. The patient continues to do well 2 years post-op with no signs of recurrence.

## 1. Introduction and Background

Biliary cystadenoma is an uncommon benign cystic neoplasm with potential for malignant transformation [1]. Preoperatively, it is difficult to distinguish biliary cystadenoma from biliary cystadenocarcinoma and hence surgical excision should be considered [2]. The majority of patients are middle-aged women with an average age of 45 at time of diagnosis. The most common symptoms are those that are due to mass effect, including epigastric and right upper quadrant (RUQ) pain, jaundice, and cholangitis [1]. A biliary cystadenoma presenting with acute pancreatitis is an uncommon presentation. This case highlights a unique presentation of biliary cystadenoma, the difficulty in identifying the origin of the mass by radiological imaging, and the appropriate use of mesohepatectomy for management of central liver neoplasms with parenchymal preserving technique to avoid postoperative liver failure (POLF) due to small future liver remnant (FLR).

## 2. Case Presentation

A 34-year-old female presented to the emergency room with upper abdominal pain and epigastric fullness. She had no associated nausea or vomiting and denied any alcohol consumption or cholecystectomy. Blood work showed elevated amylase and lipase levels consistent with pancreatitis. Serum beta-HCG was negative. She was admitted for acute pancreatitis and treated supportively with pain management and hydration. Her amylase and lipase levels returned to normal within 36 hours.

**Figure 1:**
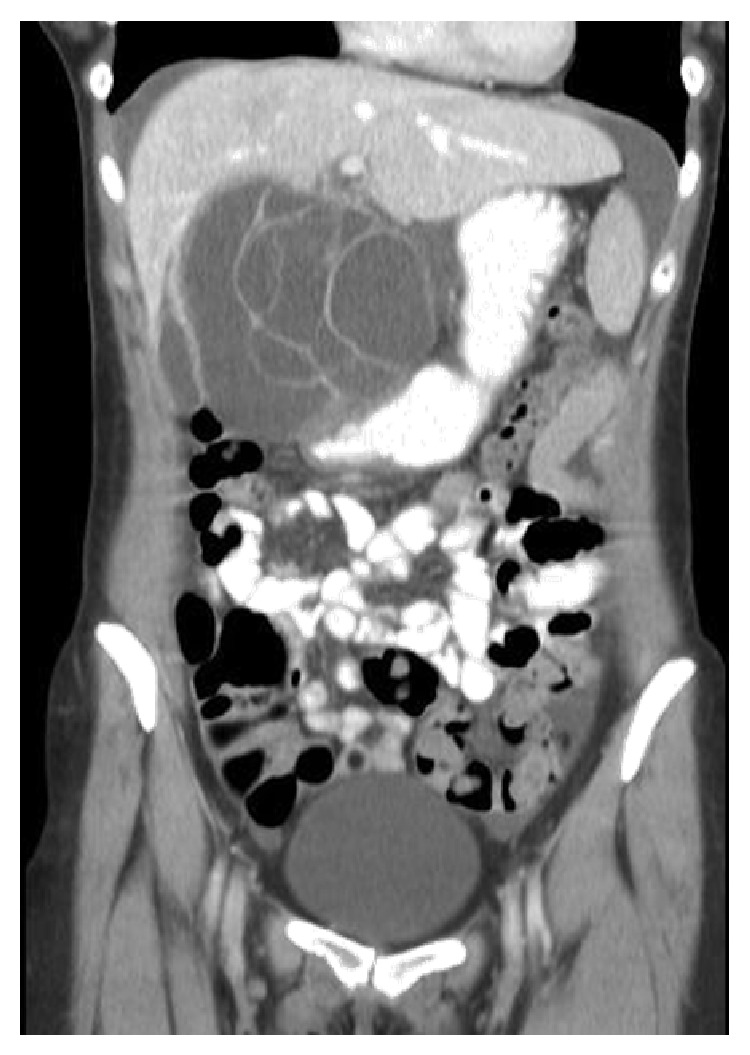
Name of image: CT coronal reformat with intravenous contrast. Description: contrast enhanced CT confirmed the presence of a large complex cystic mass. The origin of the mass was in close proximity to the pancreas, liver, and stomach. No biliary or pancreatic duct dilatation was observed on CT.

Ultrasound revealed a normal gall bladder and a large cystic mass in the epigastrium prompting further imaging. A contrast enhanced CT confirmed the presence of a large, complex cystic mass with septations measuring 15 × 15 cm in close proximity to the pancreas and extending to the liver and stomach (Figure 1). No biliary or pancreatic duct dilatation was observed. The mass was suspected to be of hepatic orpancreatic origin; however this could not be determined on CT alone.

**Figure 2:**
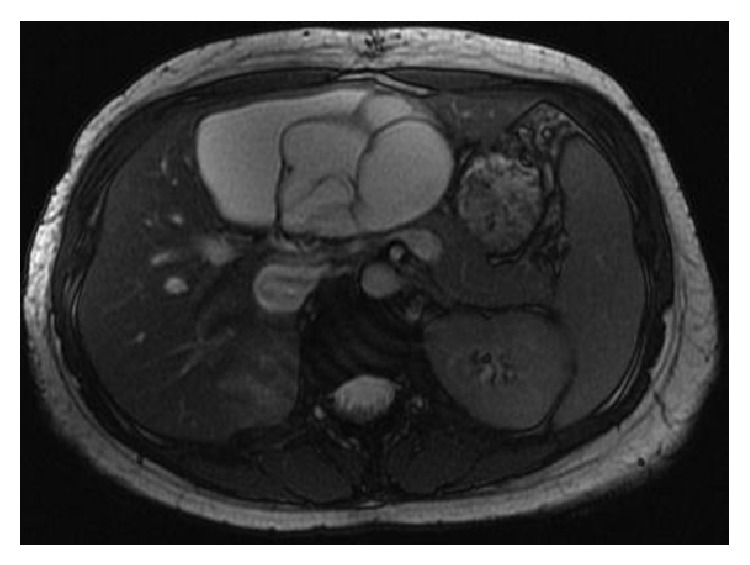
Name: axial 2D FIESTA. Description: multiple low T2 signal septations were seen within the mass on MRI.

**Figure 3:**
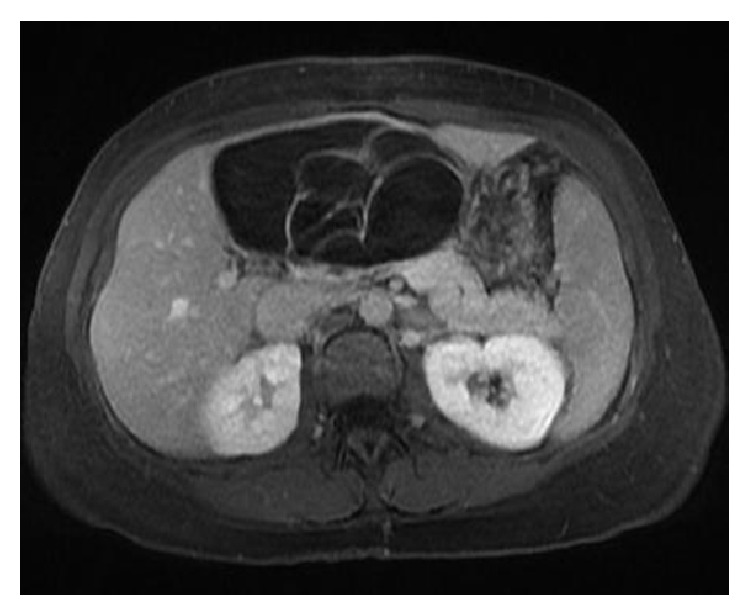
Name: axial LAVA PV following gadolinium administration in the portal venous phase. Description: enhancement of the septations was observed following the administration of gadolinium on the axial T1 image with fat saturation.

On MRI of the abdomen, the locules of the cystic mass were shown to be homogeneously T2 hyperintense and T1 hypointense with the mass overall measuring up to 14 cm in greatest dimension. The mass also demonstrated multiple low T2 signal septations measuring up to 4 mm in thickness (Figure 2) and showed enhancement following gadolinium administration (Figure 3). No nodules were seen. MR images favored the mass to be of hepatic origin. No upper abdominal lymphadenopathy was observed and the suspicion was raised for a biliary cystadenoma or cystadenocarcinoma prompting surgical consultation.

General surgery described a complex cystic mass on CT occupying parts of segments 3, 4a, 4b, 5, and 8 without any involvement of major vessels. Endoscopic US revealed a normal pancreas and confirmed the hepatic origin of the cystic mass. Tumor markers (CEA, AFP, and CA 19-9) were normal and the patient was sent for definitive treatment with hepatobiliary surgery.

**Figure 4:**
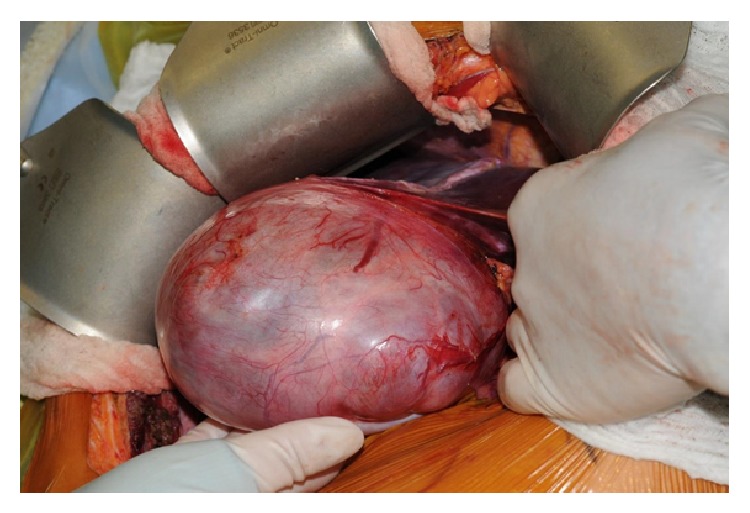
Name: mobilization of the biliary cystadenoma prior to resection. Description: the massive biliary cystadenoma, measuring 18 cm across, was mobilized to reveal the extent of hepatic involvement prior to resection.

**Figure 5:**
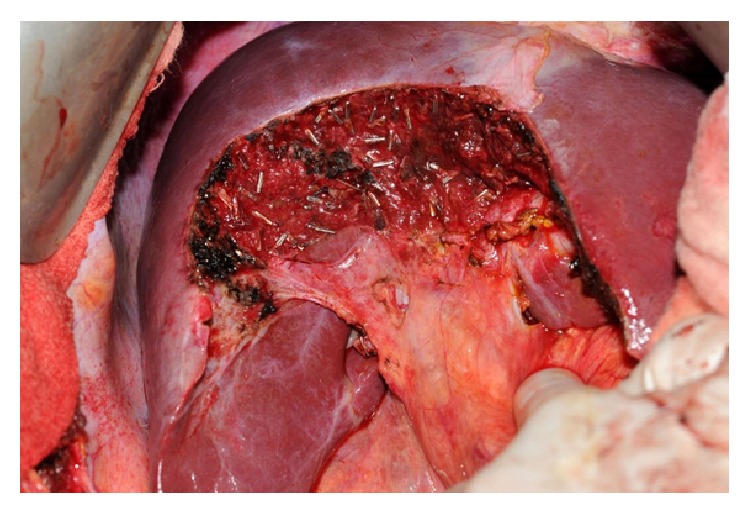
Name: postmesohepatectomy. Description: intraoperative image of the liver postresection of segments 4a, 4b, and 8, revealing preservation of the portal vasculature within the hepatoduodenal ligament.

Patient consent was obtained for a planned central hepatectomy or mesohepatectomy of segments 4a, 4b, 5, and 8 in order to preserve an adequate FLR (Figures 4 and 5). Intraoperative US was used to identify and avoid injury to the right and left hepatic veins. The transsection was performed with Conmed ALTRUS, a thermal tissue fusion system. A cholangiogram showed no evidence of a leak or injury. The patient did well in follow-up.

## 3. Discussion

Biliary cystadenomas are rare hepatic lesions that often present with nonspecific signs and symptoms [3]. Though variable, the most common presentation is asymptomatic on incidental findings through imaging [4]. To our knowledge, this is the first reported case of a biliary cystadenoma presenting as an episode of acute pancreatitis. The mass effect of the lesion on the head of the pancreas likely led to pancreatitis and early satiety in this patient. The rupture of the cyst released free fluid that was seen on imaging and likely alleviated the obstructive symptoms.

Due to both the potential for the malignant transformation of a biliary cystadenoma to cystadenocarcinoma and the inability to differentiate a benign from malignant mass preoperatively, complete surgical excision is the recommended course of treatment [5].

Biliary cystadenomas arise from the epithelium cells lining either the gall bladder or the bile ducts and are multiloculated and multiseptated. These masses typically arise from the bile ducts of the right hepatic lobe [6]. Although benign, cystadenomas can reoccur after incomplete surgical excision and may transform into malignant biliary cystadenocarcinoma or more rarely undergo sarcomatous transformation [5, 7]. Ovarian-type stroma is found in 85% of cases of biliary cystadenoma and is associated with a better prognosis should a malignant transformation occur [7].

On Ultrasound, biliary cystadenomas are typically multiloculated and demonstrate enhanced transmission. Furthermore, if septal or wall calcifications are present, acoustic shadowing may be exhibited [5]. The content of the cystic mass is usually hypoattenuating on CT [7]. On MRI, the masses are typically of low signal on T1 and high signal on T2 weighted images. Both CT attenuation and MR T1 and T2 weighted images signal intensity will vary depending on the protein content and presence of blood in the fluid component of the cystadenoma [5]. Higher CT attenuation or high T1 signal on MRI raises the possibility of recent hemorrhage [5], while it may be normal for a cystadenoma to show septal and wall enhancement on MR, if the enhancement is irregular and papillary projections are seen there should be a higher level of suspicion for malignant biliary cystadenocarcinoma [7].

Currently, the imaging modality of choice in the initial evaluation of liver masses is CT [6]. As there is no diagnostic imaging modality that reliably allows us to differentiate a benign biliary cystadenoma from a malignant biliary cystadenocarcinoma, correlation with the patient's age and clinical presentation must be taken into account when interpreting images.

Extended hepatectomy is the procedure of choice for hepatic neoplasms involving central segments of the liver 4a, 4b, 5, and 8 [8]. However, for large centrally located masses, mesohepatectomy (resection of segments 4, 5, and 8) may be preferred in order to preserve a larger standardized future liver remnant (sFLR) and avoid POLF [8].

To date, mesohepatectomy is seldom used due to the technical challenges of the procedure and risks of vascular damage owing to the anatomical complexity of the liver [8]. However, Qui et al. recently demonstrated that when compared to extended left or right hepatectomy in over 400 patients, mesohepatectomy was associated with fewer intraoperative and postoperative complications [9].

Advances in surgical techniques, such as intraoperative imaging and hemostatic transection devices, are encouraging the use of mesohepatectomy in select patient populations [10]. This case demonstrates the utility of mesohepatectomy for neoplasms of the central segments in limiting parenchymal loss and maintaining functional anatomy.
